# 2-(4-Chloro-2-nitro­phen­yl)-9-phenyl­sulfonyl-9*H*-carbazole-3-carbaldehyde

**DOI:** 10.1107/S1600536813022253

**Published:** 2013-08-14

**Authors:** S. Gopinath, K. Sethusankar, Bose Muthu Ramalingam, Arasambattu K. Mohanakrishnan

**Affiliations:** a Department of Physics, RKM Vivekananda College (Autonomous), Chennai 600 004, India; b Department of Organic Chemistry, University of Madras, Guindy Campus, Chennai 600 025, India

## Abstract

In the title compound, C_25_H_15_ClN_2_O_6_S, the carbazole ring system is essentially planar, with a maximum deviation of 0.152 (3) Å for the C atom to which the 4-chloro-2-nitro­phenyl ring is attached. Its mean plane is almost orthogonal to the phenyl­sulfonyl and nitro­phenyl rings, making dihedral angles of 82.64 (14) and 79.89 (13)°, respectively. The N atom of the nitro group deviates by 0.032 (3) Å from the benzene ring to which it is attached. The mol­ecular structure features intra­molecular O—H⋯O and C—H⋯O hydrogen bonds, which generate three *S*(6) ring motifs. In the crystal, mol­ecules are linked by C—H⋯O hydrogen bonds, which generate *C*(6) and *C*(9) chains running in the [100] and [010] directions, respectively, forming a two-dimensional network lying parallel to (001). There are also *R*_4_^3^(28) supra­molecular graph-set ring motifs enclosed within these networks.

## Related literature

For the biological activity and uses of carbazole derivatives, see: Itoigawa *et al.* (2000 ▶); Ramsewak *et al.* (1999 ▶). For their electronic properties and applications, see: Friend *et al.* (1999 ▶); Zhang *et al.* (2004 ▶). For related structures, see: Chakkaravarthi *et al.* (2008 ▶). For bond-length distortions, see: Allen *et al.* (1987 ▶). For graph-set notation, see: Bernstein *et al.* (1995 ▶). For the Thorpe–Ingold effect, see: Bassindale *et al.* (1984 ▶).
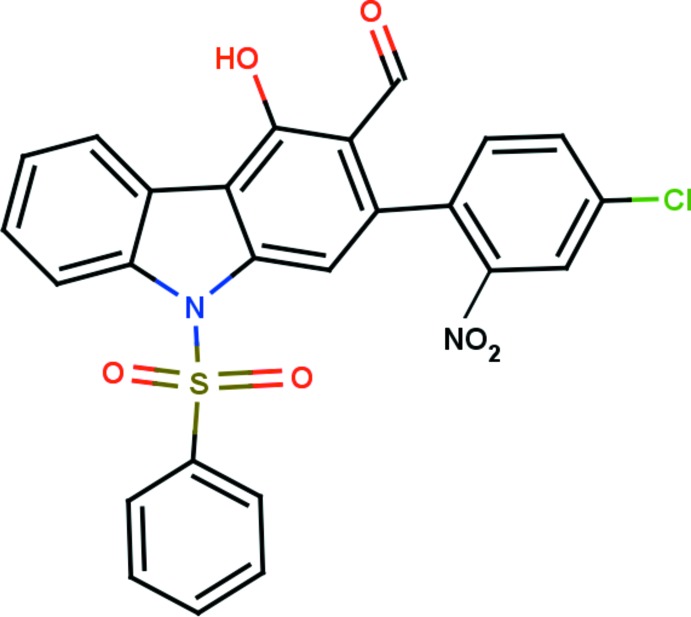


## Experimental

### 

#### Crystal data

C_25_H_15_ClN_2_O_6_S
*M* *_r_* = 506.91
Monoclinic, 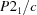

*a* = 8.1947 (15) Å
*b* = 14.384 (3) Å
*c* = 18.795 (3) Å
β = 90.963 (9)°
*V* = 2215.2 (7) Å^3^
*Z* = 4
Mo *K*α radiation
μ = 0.31 mm^−1^
*T* = 296 K
0.30 × 0.25 × 0.20 mm

#### Data collection

Bruker SMART APEXII CCD diffractometer
Absorption correction: multi-scan (*SADABS*; Bruker, 2008 ▶) *T*_min_ = 0.910, *T*_max_ = 0.939
16586 measured reflections
3893 independent reflections
2809 reflections with *I* > 2σ(*I*)
*R*_int_ = 0.056

#### Refinement

*R*[*F* ^2^ > 2σ(*F* ^2^)] = 0.053
*wR*(*F* ^2^) = 0.172
*S* = 1.05
3893 reflections
316 parameters
H-atom parameters constrained
Δρ_max_ = 0.42 e Å^−3^
Δρ_min_ = −0.40 e Å^−3^

### 

Data collection: *APEX2* (Bruker, 2008 ▶); cell refinement: *SAINT* (Bruker, 2008 ▶); data reduction: *SAINT*; program(s) used to solve structure: *SHELXS97* (Sheldrick, 2008 ▶); program(s) used to refine structure: *SHELXL97* (Sheldrick, 2008 ▶); molecular graphics: *ORTEP-3 for Windows* (Farrugia, 2012 ▶) and *Mercury* (Macrae *et al.*, 2008 ▶); software used to prepare material for publication: *SHELXL97* and *PLATON* (Spek, 2009 ▶).

## Supplementary Material

Crystal structure: contains datablock(s) global, I. DOI: 10.1107/S1600536813022253/su2632sup1.cif

Structure factors: contains datablock(s) I. DOI: 10.1107/S1600536813022253/su2632Isup2.hkl

Supplementary material file. DOI: 10.1107/S1600536813022253/su2632Isup3.cml

Additional supplementary materials:  crystallographic information; 3D view; checkCIF report

## Figures and Tables

**Table 1:** Hydrogen-bond geometry (Å, °)

*D*—H⋯*A*	*D*—H	H⋯*A*	*D*⋯*A*	*D*—H⋯*A*
O1—H1⋯O2	0.82	1.91	2.629 (4)	146
C2—H2⋯O3	0.93	2.36	2.949 (5)	121
C9—H9⋯O4	0.93	2.31	2.910 (4)	122
C13—H13⋯O4^i^	0.93	2.56	3.411 (4)	153
C18—H18⋯O4^ii^	0.93	2.53	3.287 (4)	139

Symmetry codes: (i) 
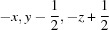
; (ii) 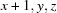.

**Fig. 1.**
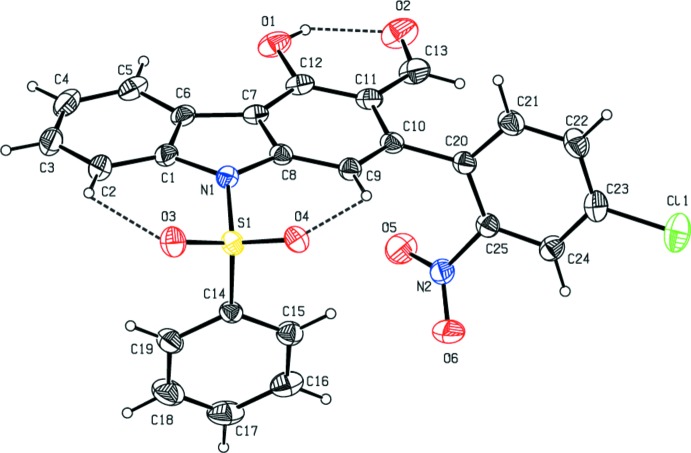
The molecular structure of the title molecule, with atom labelling. Displacement ellipsoids are drawn at 30% probability level. The intramolecular O-H···O and C-H···O hydrogen bonds are shown as dashed lines (see Table 1 for details).

**Fig. 2.**
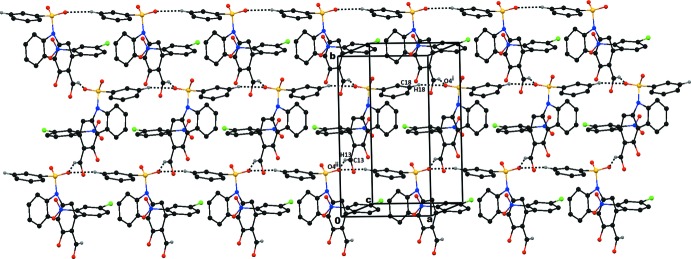
The crystal packing of the title compound viewed along the c axis. The dashed lines indicate C—H···O hydrogen bonds [see Table 1 for details; symmetry codes: (i) x+1, y, z; (ii) -x, y-1/2, -z+1/2].

## supplementary crystallographic information

### 1. Comment

Carbazole and its derivative have become quite attractive compounds owing to their applications in pharmacy and molecular electronics. It has been reported that carbazole derivatives exhibit various biological activities such as antitumor and antioxidative (Itoigawa *et al.*, 2000), and anti-inflammatory and antimutagenic (Ramsewak *et al.*, 1999). They also exhibit electroactivity and luminenscence and are considered to be potential candidates for electronic applications such as colour displays, organic, semiconductors, laser and solar cells (Friend *et al.*, 1999; Zhang *et al.*, 2004).

The title compound, Fig. 1, comprises a carbazole ring system which is attached to a phenyl-sulfonyl ring, a chloro substituted nitro-phenyl ring, a carbaldehyde group and a hydroxyl group. The carbazole ring system is essentially planar with maximum deviation of -0.152 (3) Å for atom C10 to which is attached the 4-chloro-2-nitro-phenyl ring. Atom O1 significantly deviates from the carbazole ring mean plane by -0.1921 (24) Å. The carbazole ring system is almost orthogonal to the phenyl ring attached to sulfonyl group and to the 4-chloro-2-nitro-phenyl ring with dihedral angles of 82.64 (14)° and 79.89 (13)°, respectively.

As a result of electron-withdrawing character of the phenylsulfonyl group the bond lengths N1–C1 = 1.429 (4) Å and N1–C8 = 1.408 (4) Å are longer than the mean value of 1.355 (14) Å (Allen *et al.*, 1987). Atom S1 has a distorted tetrahedral configuration. The widening of angle O3—S1—O4 [120.14 (14) °] and the narrowing of angle N1—S1—C11 [103.36 (13)°] from the ideal tetrahedral value are attributed to the Thorpe-Ingold effect (Bassindale *et al.*, 1984).

The sum of the bond angles around atom N1 [355.1°] indicate sp^2^ hybridization. Atom N2 and chlorine atom Cl1 deviate significantly, by 0.0319 (31) Å and -0.0240 (13) Å, respectively, from the phenyl ring.

The molecular structure is stabilized by O-H···O and C-H···O hydrogen bonds (Table 1 and Fig. 1), which generate three S(6) ring motifs (Bernstein *et al.*, 1995).

In the crystal, molecules are linked by C-H···O hydrogen bonds, which generate *C*(6) and *C*(9) chains running in directions [100] and [010], respectively (Table 1 and Fig. 2), and forming a two-dimensional network lying parallel to (001). There are also *R*^3^_4_(28) supramolecular graph-set ring motifs enclosed within these networks.

### 2. Experimental

The enamine, (2E)-1-[1-(phenylsulfonyl)-2-[(E)-2-(4-cholro-2-nitrophenyl)ethenyl]-2,3 -dihydro-1H-indol-3-yl]-3-dimethylamino)prop-2-en-1-one (0.500 g, 0.93 mmol), with CuBr_2_ (0.209 g, 0.93 mmol) in dry DMF (20 ml) was refluxed under N_2_ for 1 h. The mixture was then poured over crushed ice (50 g) containing conc. HCl (1 ml). The crude product was filtered and dried. It was then purified by flash column chromatography on silica gel (230–420 mesh, n-hexane/ethyl acetate, 7:3) to afford the title compound as a pale yellow solid [Yield= 91%]. Yellow block-like crystal were obtained by slow evaporation of a solution in CHCl_3_.

### 3. Refinement

The positions of all the H atoms were located in difference electron density maps. They were refined as riding atoms: O—H = = 0.82 Å and C-H = 0.93 Å with *U*iso(H) = 1.5*U*eq(O-hydroxyl) and = 1.2Ueq(C-aromatic).

### Crystal data

**Table:**

C_25_H_15_ClN_2_O_6_S	*F*(000) = 1040
*M**_r_* = 506.91	*D*_x_ = 1.520 Mg m^−^^3^
Monoclinic, *P*2_1_/*c*	Mo *K*α radiation, λ = 0.71073 Å
Hall symbol: -P 2ybc	Cell parameters from 3893 reflections
*a* = 8.1947 (15) Å	θ = 1.8–25.0°
*b* = 14.384 (3) Å	µ = 0.31 mm^−^^1^
*c* = 18.795 (3) Å	*T* = 296 K
β = 90.963 (9)°	Block, yellow
*V* = 2215.2 (7) Å^3^	0.30 × 0.25 × 0.20 mm
*Z* = 4	

### Data collection

**Table:**

Bruker SMART APEXII CCD diffractometer	3893 independent reflections
Radiation source: fine-focus sealed tube	2809 reflections with *I* > 2σ(*I*)
Graphite monochromator	*R*_int_ = 0.056
ω & φ scans	θ_max_ = 25.0°, θ_min_ = 1.8°
Absorption correction: multi-scan (*SADABS*; Bruker, 2008)	*h* = −9→9
*T*_min_ = 0.910, *T*_max_ = 0.939	*k* = −17→15
16586 measured reflections	*l* = −22→22

### Refinement

**Table:**

Refinement on *F*^2^	Primary atom site location: structure-invariant direct methods
Least-squares matrix: full	Secondary atom site location: difference Fourier map
*R*[*F*^2^ > 2σ(*F*^2^)] = 0.053	Hydrogen site location: inferred from neighbouring sites
*wR*(*F*^2^) = 0.172	H-atom parameters constrained
*S* = 1.05	*w* = 1/[σ^2^(*F*_o_^2^) + (0.0933*P*)^2^ + 0.9326*P*] where *P* = (*F*_o_^2^ + 2*F*_c_^2^)/3
3893 reflections	(Δ/σ)_max_ < 0.001
316 parameters	Δρ_max_ = 0.42 e Å^−^^3^
0 restraints	Δρ_min_ = −0.40 e Å^−^^3^

### Special details

**Table:**

Geometry. All e.s.d.'s (except the e.s.d. in the dihedral angle between two l.s. planes) are estimated using the full covariance matrix. The cell e.s.d.'s are taken into account individually in the estimation of e.s.d.'s in distances, angles and torsion angles; correlations between e.s.d.'s in cell parameters are only used when they are defined by crystal symmetry. An approximate (isotropic) treatment of cell e.s.d.'s is used for estimating e.s.d.'s involving l.s. planes.
Refinement. Refinement of *F*^2^ against ALL reflections. The weighted *R*-factor *wR* and goodness of fit *S* are based on *F*^2^, conventional *R*-factors *R* are based on *F*, with *F* set to zero for negative *F*^2^. The threshold expression of *F*^2^ > σ(*F*^2^) is used only for calculating *R*-factors(gt) *etc*. and is not relevant to the choice of reflections for refinement. *R*-factors based on *F*^2^ are statistically about twice as large as those based on *F*, and *R*- factors based on ALL data will be even larger.

### Fractional atomic coordinates and isotropic or equivalent isotropic displacement parameters (Å^2^)

**Table:**

	*x*	*y*	*z*	*U*_iso_*/*U*_eq_	
C1	0.2551 (3)	0.6180 (2)	0.50926 (15)	0.0528 (8)	
C2	0.3370 (4)	0.6615 (3)	0.56478 (17)	0.0671 (9)	
H2	0.3374	0.7259	0.5694	0.081*	
C3	0.4184 (4)	0.6050 (4)	0.61323 (19)	0.0788 (12)	
H3	0.4772	0.6323	0.6505	0.095*	
C4	0.4150 (5)	0.5097 (4)	0.60796 (19)	0.0780 (11)	
H4	0.4717	0.4740	0.6414	0.094*	
C5	0.3290 (4)	0.4662 (3)	0.55391 (19)	0.0685 (9)	
H5	0.3241	0.4017	0.5512	0.082*	
C6	0.2497 (3)	0.5214 (2)	0.50362 (16)	0.0536 (8)	
C7	0.1606 (3)	0.4993 (2)	0.43898 (16)	0.0505 (7)	
C8	0.1173 (3)	0.5826 (2)	0.40603 (16)	0.0469 (7)	
C9	0.0421 (3)	0.5859 (2)	0.33864 (16)	0.0494 (7)	
H9	0.0132	0.6421	0.3175	0.059*	
C10	0.0132 (3)	0.5029 (2)	0.30519 (16)	0.0501 (7)	
C11	0.0462 (4)	0.4168 (2)	0.33792 (18)	0.0561 (8)	
C12	0.1204 (4)	0.4155 (2)	0.40598 (18)	0.0569 (8)	
C13	0.0128 (5)	0.3305 (3)	0.3022 (2)	0.0770 (11)	
H13	−0.0318	0.3342	0.2565	0.092*	
C14	0.3698 (3)	0.7616 (2)	0.37430 (17)	0.0493 (7)	
C15	0.3674 (4)	0.7328 (2)	0.3047 (2)	0.0622 (9)	
H15	0.2690	0.7201	0.2813	0.075*	
C16	0.5117 (5)	0.7232 (3)	0.2703 (2)	0.0809 (11)	
H16	0.5125	0.7035	0.2232	0.097*	
C17	0.6562 (5)	0.7428 (3)	0.3057 (3)	0.0918 (14)	
H17	0.7546	0.7355	0.2824	0.110*	
C18	0.6566 (5)	0.7726 (3)	0.3743 (3)	0.0935 (14)	
H18	0.7549	0.7870	0.3971	0.112*	
C19	0.5137 (4)	0.7815 (3)	0.4098 (2)	0.0722 (10)	
H19	0.5135	0.8007	0.4571	0.087*	
C20	−0.0564 (3)	0.5080 (2)	0.23145 (16)	0.0503 (7)	
C21	−0.2215 (4)	0.5008 (3)	0.21838 (19)	0.0681 (9)	
H21	−0.2892	0.4855	0.2558	0.082*	
C22	−0.2902 (4)	0.5152 (3)	0.1522 (2)	0.0718 (10)	
H22	−0.4023	0.5092	0.1451	0.086*	
C23	−0.1919 (4)	0.5385 (3)	0.09668 (19)	0.0658 (9)	
C24	−0.0268 (4)	0.5468 (2)	0.10637 (17)	0.0592 (8)	
H24	0.0399	0.5635	0.0689	0.071*	
C25	0.0376 (3)	0.5298 (2)	0.17300 (16)	0.0519 (7)	
Cl1	−0.27788 (15)	0.55884 (9)	0.01388 (6)	0.1018 (4)	
N1	0.1664 (3)	0.65682 (17)	0.45026 (13)	0.0503 (6)	
N2	0.2158 (3)	0.5375 (2)	0.18092 (15)	0.0664 (8)	
O1	0.1583 (3)	0.33589 (17)	0.43974 (14)	0.0784 (7)	
H1	0.1286	0.2916	0.4153	0.118*	
O2	0.0370 (5)	0.25321 (19)	0.32590 (17)	0.1032 (10)	
O3	0.2092 (3)	0.82164 (17)	0.48325 (13)	0.0717 (7)	
O4	0.0573 (2)	0.78288 (15)	0.37344 (12)	0.0569 (6)	
O5	0.2857 (3)	0.4869 (2)	0.22158 (17)	0.0960 (9)	
O6	0.2834 (3)	0.5962 (3)	0.14560 (17)	0.1149 (12)	
S1	0.18862 (9)	0.76577 (5)	0.42149 (4)	0.0504 (3)	

### Atomic displacement parameters (Å^2^)

**Table:**

	*U*^11^	*U*^22^	*U*^33^	*U*^12^	*U*^13^	*U*^23^
C1	0.0501 (15)	0.063 (2)	0.0456 (16)	−0.0008 (13)	0.0061 (13)	0.0019 (15)
C2	0.071 (2)	0.081 (3)	0.0489 (19)	−0.0099 (18)	0.0001 (16)	−0.0027 (17)
C3	0.071 (2)	0.116 (4)	0.049 (2)	−0.006 (2)	−0.0034 (17)	0.004 (2)
C4	0.076 (2)	0.108 (4)	0.051 (2)	0.013 (2)	0.0055 (18)	0.022 (2)
C5	0.073 (2)	0.077 (2)	0.057 (2)	0.0105 (18)	0.0189 (17)	0.0186 (19)
C6	0.0520 (16)	0.065 (2)	0.0445 (16)	−0.0013 (14)	0.0122 (13)	0.0055 (15)
C7	0.0482 (15)	0.0523 (19)	0.0514 (17)	−0.0029 (13)	0.0147 (13)	0.0056 (15)
C8	0.0425 (13)	0.0495 (18)	0.0490 (16)	−0.0033 (12)	0.0080 (12)	−0.0036 (14)
C9	0.0498 (15)	0.0492 (18)	0.0492 (17)	−0.0020 (13)	0.0025 (13)	−0.0023 (14)
C10	0.0496 (15)	0.0516 (19)	0.0494 (17)	−0.0076 (13)	0.0112 (13)	−0.0078 (15)
C11	0.0650 (18)	0.0476 (19)	0.0564 (19)	−0.0101 (14)	0.0183 (15)	−0.0057 (15)
C12	0.0645 (18)	0.048 (2)	0.0587 (19)	−0.0016 (14)	0.0212 (15)	0.0053 (16)
C13	0.106 (3)	0.059 (2)	0.068 (2)	−0.011 (2)	0.024 (2)	−0.0087 (19)
C14	0.0473 (15)	0.0424 (17)	0.0580 (18)	−0.0019 (12)	−0.0019 (13)	0.0068 (14)
C15	0.0558 (17)	0.062 (2)	0.069 (2)	0.0010 (15)	0.0040 (16)	0.0054 (17)
C16	0.080 (3)	0.083 (3)	0.080 (3)	0.008 (2)	0.025 (2)	0.015 (2)
C17	0.059 (2)	0.099 (3)	0.118 (4)	0.007 (2)	0.030 (2)	0.031 (3)
C18	0.051 (2)	0.109 (4)	0.121 (4)	−0.011 (2)	−0.002 (2)	0.025 (3)
C19	0.0564 (18)	0.077 (3)	0.083 (3)	−0.0145 (16)	−0.0066 (17)	0.010 (2)
C20	0.0544 (15)	0.0457 (18)	0.0511 (17)	−0.0051 (13)	0.0080 (13)	−0.0111 (14)
C21	0.0545 (17)	0.083 (3)	0.068 (2)	−0.0100 (16)	0.0110 (16)	−0.0126 (19)
C22	0.0532 (18)	0.084 (3)	0.078 (3)	0.0019 (16)	−0.0033 (18)	−0.017 (2)
C23	0.070 (2)	0.060 (2)	0.067 (2)	0.0141 (16)	−0.0138 (18)	−0.0133 (18)
C24	0.0674 (19)	0.058 (2)	0.0519 (19)	0.0037 (15)	0.0072 (15)	−0.0045 (15)
C25	0.0532 (16)	0.0508 (19)	0.0519 (18)	−0.0011 (13)	0.0042 (14)	−0.0087 (14)
Cl1	0.1052 (8)	0.1192 (10)	0.0800 (7)	0.0313 (7)	−0.0284 (6)	−0.0017 (6)
N1	0.0544 (13)	0.0517 (16)	0.0449 (13)	−0.0047 (11)	0.0020 (11)	−0.0015 (12)
N2	0.0553 (15)	0.093 (2)	0.0514 (16)	−0.0074 (15)	0.0070 (13)	−0.0066 (16)
O1	0.1124 (19)	0.0498 (15)	0.0736 (16)	0.0026 (13)	0.0212 (14)	0.0123 (12)
O2	0.174 (3)	0.0460 (17)	0.091 (2)	−0.0101 (17)	0.026 (2)	−0.0035 (15)
O3	0.0860 (15)	0.0605 (15)	0.0685 (15)	−0.0006 (12)	−0.0026 (12)	−0.0224 (12)
O4	0.0465 (10)	0.0543 (14)	0.0696 (14)	0.0067 (9)	−0.0036 (10)	−0.0058 (11)
O5	0.0603 (14)	0.134 (3)	0.093 (2)	0.0160 (15)	0.0053 (14)	0.0219 (19)
O6	0.0769 (17)	0.171 (3)	0.097 (2)	−0.045 (2)	0.0073 (16)	0.041 (2)
S1	0.0506 (4)	0.0452 (5)	0.0552 (5)	0.0003 (3)	−0.0015 (3)	−0.0074 (3)

### Geometric parameters (Å, º)

**Table:**

C1—C2	1.381 (4)	C14—S1	1.743 (3)
C1—C6	1.394 (5)	C15—C16	1.364 (5)
C1—N1	1.429 (4)	C15—H15	0.9300
C2—C3	1.384 (5)	C16—C17	1.378 (6)
C2—H2	0.9300	C16—H16	0.9300
C3—C4	1.375 (6)	C17—C18	1.359 (7)
C3—H3	0.9300	C17—H17	0.9300
C4—C5	1.377 (5)	C18—C19	1.364 (6)
C4—H4	0.9300	C18—H18	0.9300
C5—C6	1.388 (5)	C19—H19	0.9300
C5—H5	0.9300	C20—C21	1.374 (4)
C6—C7	1.442 (4)	C20—C25	1.388 (4)
C7—C12	1.392 (5)	C21—C22	1.373 (5)
C7—C8	1.392 (4)	C21—H21	0.9300
C8—C9	1.400 (4)	C22—C23	1.370 (5)
C8—N1	1.408 (4)	C22—H22	0.9300
C9—C10	1.369 (4)	C23—C24	1.367 (5)
C9—H9	0.9300	C23—Cl1	1.723 (4)
C10—C11	1.406 (4)	C24—C25	1.373 (4)
C10—C20	1.492 (4)	C24—H24	0.9300
C11—C12	1.407 (5)	C25—N2	1.469 (4)
C11—C13	1.435 (5)	N1—S1	1.669 (3)
C12—O1	1.343 (4)	N2—O5	1.196 (4)
C13—O2	1.213 (5)	N2—O6	1.213 (4)
C13—H13	0.9300	O1—H1	0.8200
C14—C15	1.372 (5)	O3—S1	1.420 (2)
C14—C19	1.375 (4)	O4—S1	1.415 (2)
			
C2—C1—C6	121.6 (3)	C14—C15—H15	120.5
C2—C1—N1	130.1 (3)	C15—C16—C17	119.7 (4)
C6—C1—N1	108.4 (3)	C15—C16—H16	120.2
C1—C2—C3	117.0 (4)	C17—C16—H16	120.2
C1—C2—H2	121.5	C18—C17—C16	120.7 (4)
C3—C2—H2	121.5	C18—C17—H17	119.6
C4—C3—C2	122.0 (4)	C16—C17—H17	119.6
C4—C3—H3	119.0	C17—C18—C19	120.4 (4)
C2—C3—H3	119.0	C17—C18—H18	119.8
C3—C4—C5	121.0 (4)	C19—C18—H18	119.8
C3—C4—H4	119.5	C18—C19—C14	118.6 (4)
C5—C4—H4	119.5	C18—C19—H19	120.7
C4—C5—C6	118.0 (4)	C14—C19—H19	120.7
C4—C5—H5	121.0	C21—C20—C25	115.7 (3)
C6—C5—H5	121.0	C21—C20—C10	121.5 (3)
C5—C6—C1	120.3 (3)	C25—C20—C10	122.5 (3)
C5—C6—C7	132.2 (3)	C22—C21—C20	122.6 (3)
C1—C6—C7	107.4 (3)	C22—C21—H21	118.7
C12—C7—C8	119.3 (3)	C20—C21—H21	118.7
C12—C7—C6	132.8 (3)	C23—C22—C21	119.3 (3)
C8—C7—C6	107.8 (3)	C23—C22—H22	120.4
C7—C8—C9	122.5 (3)	C21—C22—H22	120.4
C7—C8—N1	108.8 (3)	C24—C23—C22	120.9 (3)
C9—C8—N1	128.7 (3)	C24—C23—Cl1	119.6 (3)
C10—C9—C8	117.1 (3)	C22—C23—Cl1	119.5 (3)
C10—C9—H9	121.4	C23—C24—C25	118.1 (3)
C8—C9—H9	121.4	C23—C24—H24	121.0
C9—C10—C11	122.4 (3)	C25—C24—H24	121.0
C9—C10—C20	116.4 (3)	C24—C25—C20	123.5 (3)
C11—C10—C20	121.2 (3)	C24—C25—N2	116.5 (3)
C10—C11—C12	119.1 (3)	C20—C25—N2	120.1 (3)
C10—C11—C13	121.5 (3)	C8—N1—C1	107.4 (2)
C12—C11—C13	119.3 (3)	C8—N1—S1	123.5 (2)
O1—C12—C7	118.5 (3)	C1—N1—S1	124.1 (2)
O1—C12—C11	122.2 (3)	O5—N2—O6	123.7 (3)
C7—C12—C11	119.3 (3)	O5—N2—C25	118.9 (3)
O2—C13—C11	126.4 (4)	O6—N2—C25	117.4 (3)
O2—C13—H13	116.8	C12—O1—H1	109.5
C11—C13—H13	116.8	O4—S1—O3	120.14 (14)
C15—C14—C19	121.6 (3)	O4—S1—N1	106.50 (13)
C15—C14—S1	119.8 (2)	O3—S1—N1	106.18 (14)
C19—C14—S1	118.5 (3)	O4—S1—C14	109.06 (14)
C16—C15—C14	119.0 (3)	O3—S1—C14	110.19 (15)
C16—C15—H15	120.5	N1—S1—C14	103.37 (13)
			
C6—C1—C2—C3	2.3 (5)	C15—C14—C19—C18	0.3 (5)
N1—C1—C2—C3	−177.4 (3)	S1—C14—C19—C18	175.9 (3)
C1—C2—C3—C4	−1.8 (5)	C9—C10—C20—C21	−95.9 (4)
C2—C3—C4—C5	−0.3 (6)	C11—C10—C20—C21	83.9 (4)
C3—C4—C5—C6	1.9 (5)	C9—C10—C20—C25	77.3 (4)
C4—C5—C6—C1	−1.4 (5)	C11—C10—C20—C25	−102.9 (4)
C4—C5—C6—C7	175.0 (3)	C25—C20—C21—C22	−0.8 (5)
C2—C1—C6—C5	−0.7 (4)	C10—C20—C21—C22	172.9 (3)
N1—C1—C6—C5	179.0 (3)	C20—C21—C22—C23	−0.6 (6)
C2—C1—C6—C7	−177.9 (3)	C21—C22—C23—C24	0.5 (6)
N1—C1—C6—C7	1.8 (3)	C21—C22—C23—Cl1	−178.7 (3)
C5—C6—C7—C12	1.8 (5)	C22—C23—C24—C25	0.9 (5)
C1—C6—C7—C12	178.6 (3)	Cl1—C23—C24—C25	−179.8 (3)
C5—C6—C7—C8	−175.4 (3)	C23—C24—C25—C20	−2.5 (5)
C1—C6—C7—C8	1.4 (3)	C23—C24—C25—N2	178.8 (3)
C12—C7—C8—C9	−3.6 (4)	C21—C20—C25—C24	2.4 (5)
C6—C7—C8—C9	174.1 (2)	C10—C20—C25—C24	−171.2 (3)
C12—C7—C8—N1	178.3 (2)	C21—C20—C25—N2	−178.9 (3)
C6—C7—C8—N1	−4.0 (3)	C10—C20—C25—N2	7.5 (5)
C7—C8—C9—C10	−0.5 (4)	C7—C8—N1—C1	5.1 (3)
N1—C8—C9—C10	177.2 (2)	C9—C8—N1—C1	−172.9 (3)
C8—C9—C10—C11	4.2 (4)	C7—C8—N1—S1	161.01 (19)
C8—C9—C10—C20	−175.9 (2)	C9—C8—N1—S1	−16.9 (4)
C9—C10—C11—C12	−3.7 (4)	C2—C1—N1—C8	175.5 (3)
C20—C10—C11—C12	176.4 (3)	C6—C1—N1—C8	−4.2 (3)
C9—C10—C11—C13	179.1 (3)	C2—C1—N1—S1	19.7 (4)
C20—C10—C11—C13	−0.7 (4)	C6—C1—N1—S1	−160.0 (2)
C8—C7—C12—O1	−177.8 (3)	C24—C25—N2—O5	−145.9 (3)
C6—C7—C12—O1	5.2 (5)	C20—C25—N2—O5	35.2 (5)
C8—C7—C12—C11	4.0 (4)	C24—C25—N2—O6	34.5 (5)
C6—C7—C12—C11	−173.0 (3)	C20—C25—N2—O6	−144.3 (3)
C10—C11—C12—O1	−178.7 (3)	C8—N1—S1—O4	40.4 (2)
C13—C11—C12—O1	−1.5 (5)	C1—N1—S1—O4	−167.7 (2)
C10—C11—C12—C7	−0.5 (4)	C8—N1—S1—O3	169.5 (2)
C13—C11—C12—C7	176.7 (3)	C1—N1—S1—O3	−38.5 (3)
C10—C11—C13—O2	179.9 (4)	C8—N1—S1—C14	−74.5 (2)
C12—C11—C13—O2	2.8 (6)	C1—N1—S1—C14	77.5 (2)
C19—C14—C15—C16	0.5 (5)	C15—C14—S1—O4	−29.1 (3)
S1—C14—C15—C16	−175.0 (3)	C19—C14—S1—O4	155.3 (3)
C14—C15—C16—C17	−0.3 (6)	C15—C14—S1—O3	−162.9 (3)
C15—C16—C17—C18	−0.7 (6)	C19—C14—S1—O3	21.4 (3)
C16—C17—C18—C19	1.6 (7)	C15—C14—S1—N1	84.0 (3)
C17—C18—C19—C14	−1.3 (6)	C19—C14—S1—N1	−91.7 (3)

### Hydrogen-bond geometry (Å, º)

**Table:**

*D*—H···*A*	*D*—H	H···*A*	*D*···*A*	*D*—H···*A*
O1—H1···O2	0.82	1.91	2.629 (4)	146
C2—H2···O3	0.93	2.36	2.949 (5)	121
C9—H9···O4	0.93	2.31	2.910 (4)	122
C13—H13···O4^i^	0.93	2.56	3.411 (4)	153
C18—H18···O4^ii^	0.93	2.53	3.287 (4)	139

Symmetry codes: (i) −*x*, *y*−1/2, −*z*+1/2; (ii) *x*+1, *y*, *z*.
